# Supplementary material for: The Use of Non-Nutritive and Low-Calorie Sweeteners in 19,915 Local and Imported Pre-Packaged Foods in Hong Kong
Source: Nutrients. 2021 May 29;13(6):1861. doi: 10.3390/nu13061861 (PMC8229473; doi:10.3390/nu13061861)
Supplement: Supplementary file 1 [file nutrients-13-01861-s001.zip › nutrients-1209616-supplementary.pdf]

Supplementary Table 1. – Prevalence [*n* (%)] of use of different non-nutritive sweeteners (NNSs) by food category[1].

Food category	Total *n* in category	E950	E951	E952	E954	E955	E959	E960	E961	E962	E969	Contains at least one NNS
Bread and bakery products	1774	5 (0.3)	1 (0.1)	0 (0.0)	5 (0.3)	15 (0.8)	0 (0.0)	3 (0.2)	1 (0.1)	0 (0.0)	0 (0.0)	24 (1.4)
Biscuits	1085	5 (0.5)	1 (0.1)	0 (0.0)	4 (0.4)	14 (0.3)	0 (0.0)	2 (0.2)	1 (0.1)	0 (0.0)	0 (0.0)	21 (1.9)
Breads	181	0 (0.0)	0 (0.0)	0 (0.0)	1 (0.6)	0 (0.0)	0 (0.0)	0 (0.0)	0 (0.0)	0 (0.0)	0 (0.0)	1 (0.6)
Cakes and other bakery items	508	0 (0.0)	0 (0.0)	0 (0.0)	0 (0.0)	1 (0.2)	0 (0.0)	1 (0.2)	0 (0.0)	0 (0.0)	0 (0.0)	2 (0.4)
Cereal and grain products	2826	16 (0.6)	1 (0.0)	0 (0.0)	0 (0.0)	20 (0.7)	0 (0.0)	23 (0.8)	0 (0.0)	0 (0.0)	1 (0.0)	44 (1.6)
Breakfast cereals	466	0 (0.0)	0 (0.0)	0 (0.0)	0 (0.0)	0 (0.0)	0 (0.0)	0 (0.0)	0 (0.0)	0 (0.0)	0 (0.0)	0 (0.0)
Cereals and nut-based bars	118	0 (0.0)	0 (0.0)	0 (0.0)	0 (0.0)	0 (0.0)	0 (0.0)	0 (0.0)	0 (0.0)	0 (0.0)	0 (0.0)	0 (0.0)
Noodles	1127	16 (1.4)	0 (0.0)	0 (0.0)	0 (0.0)	20 (1.8)	0 (0.0)	23 (2.0)	0 (0.0)	0 (0.0)	1 (0.1)	43 (3.8)
Pasta, rice or cous cous	655	0 (0.0)	1 (0.2)	0 (0.0)	0 (0.0)	0 (0.0)	0 (0.0)	0 (0.0)	0 (0.0)	0 (0.0)	0 (0.0)	1 (0.2)
Other cereal products	460	0 (0.0)	0 (0.0)	0 (0.0)	0 (0.0)	0 (0.0)	0 (0.0)	0 (0.0)	0 (0.0)	0 (0.0)	0 (0.0)	0 (0.0)
Confectionery	1710	145 (8.5)	98 (5.7)	1 (0.1)	2 (0.1)	74 (4.3)	0 (0.0)	20 (1.2)	1 (0.1)	1 (0.1)	0 (0.0)	190 (11.1)
Chocolates	735	6 (0.8)	6 (0.8)	0 (0.0)	0 (0.0)	0 (0.0)	0 (0.0)	10 (1.4)	0 (0.0)	0 (0.0)	0 (0.0)	16 (2.2)
Sweets	658	44 (6.7)	22 (3.3)	0 (0.0)	0 (0.0)	36 (5.5)	0 (0.0)	8 (1.2)	0 (0.0)	0 (0.0)	0 (0.0)	63 (9.6)
Chewing gums	57	43 (75.4)	47 (82.5)	0 (0.0)	0 (0.0)	10 (17.5)	0 (0.0)	0 (0.0)	0 (0.0)	0 (0.0)	0 (0.0)	51 (89.5)
Cough lollies	61	19 (31.1)	14 (23.0)	0 (0.0)	1 (1.6)	6 (9.8)	0 (0.0)	1 (1.6)	0 (0.0)	0 (0.0)	0 (0.0)	23 (37.7)
Jellies	199	33 (16.6)	9 (4.5)	1 (0.5)	1 (0.5)	22 (11.1)	0 (0.0)	1 (0.5)	1 (0.5)	1 (0.5)	0 (0.0)	37 (18.6)
Convenience foods	1171	3 (0.3)	0 (0.0)	0 (0.0)	1 (0.1)	11 (0.9)	0 (0.0)	9 (0.8)	0 (0.0)	0 (0.0)	0 (0.0)	24 (2.0)
Dairy	1611	23 (1.4)	6 (0.4)	1 (0.1)	2 (0.1)	24 (1.5)	0 (0.0)	14 (0.9)	0 (0.0)	0 (0.0)	0 (0.0)	53 (3.3)
Edible oils and oil emulsions	459	0 (0.0)	0 (0.0)	0 (0.0)	0 (0.0)	0 (0.0)	0 (0.0)	0 (0.0)	0 (0.0)	0 (0.0)	0 (0.0)	0 (0.0)
Eggs	74	0 (0.0)	0 (0.0)	0 (0.0)	0 (0.0)	0 (0.0)	0 (0.0)	0 (0.0)	0 (0.0)	0 (0.0)	0 (0.0)	0 (0.0)
Seafood and seafood products	740	6 (0.8)	0 (0.0)	0 (0.0)	0 (0.0)	1 (0.1)	0 (0.0)	9 (1.2)	0 (0.0)	0 (0.0)	0 (0.0)	16 (2.2)
Fruit and vegetables	2525	13 (0.5)	25 (1.0)	27 (1.1)	31 (1.2)	22 (0.9)	0 (0.0)	7 (0.3)	4 (0.2)	2 (0.1)	0 (0.0)	85 (3.4)
Fruits	460	0 (0.0)	13 (2.8)	24 (5.2)	19 (4.1)	9 (2.0)	0 (0.0)	1 (0.2)	1 (0.2)	1 (0.2)	0 (0.0)	35 (7.6)
Herbs and spices	586	0 (0.0)	2 (0.3)	0 (0.0)	0 (0.0)	1 (0.2)	0 (0.0)	1 (0.2)	1 (0.2)	0 (0.0)	0 (0.0)	3 (0.5)
Jam and marmalade	222	0 (0.0)	0 (0.0)	0 (0.0)	0 (0.0)	3 (1.4)	0 (0.0)	0 (0.0)	0 (0.0)	0 (0.0)	0 (0.0)	3 (1.4)
Nuts and seeds	431	9 (2.1)	8 (1.9)	3 (0.7)	2 (0.5)	4 (0.9)	0 (0.0)	1 (0.2)	0 (0.0)	0 (0.0)	0 (0.0)	22 (5.1)
Vegetables	826	4 (0.5)	2 (0.2)	0 (0.0)	10 (1.2)	5 (0.6)	0 (0.0)	4 (0.5)	2 (0.2)	1 (0.1)	0 (0.0)	22 (2.7)
Meat and meat alternatives	763	0 (0.0)	0 (0.0)	0 (0.0)	0 (0.0)	2 (0.3)	0 (0.0)	1 (0.1)	0 (0.0)	0 (0.0)	0 (0.0)	3 (0.4)
Non-alcoholic beverages	2423	98 (4.0)	62 (2.6)	1 (0.0)	11 (0.5)	130 (5.4)	1 (0.0)	42 (1.7)	1 (0.0)	6 (0.2)	0 (0.0)	241 (9.9)
Coffee, tea, and beverage mixes	915	27 (3.0)	7 (0.8)	0 (0.0)	0 (0.0)	8 (0.9)	0 (0.0)	5 (0.5)	0 (0.0)	0 (0.0)	0 (0.0)	34 (3.7)
Cordials	94	0 (0.0)	4 (4.3)	0 (0.0)	6 (6.4)	12 (12.8)	0 (0.0)	0 (0.0)	0 (0.0)	0 (0.0)	0 (0.0)	18 (19.1)
Electrolyte and energy drinks	45	8 (17.7)	1 (2.2)	0 (0.0)	0 (0.0)	16 (35.6)	0 (0.0)	1 (2.2)	0 (0.0)	0 (0.0)	0 (0.0)	20 (44.4)
Fruit and vegetable juices	527	2 (0.4)	1 (0.2)	0 (0.0)	0 (0.0)	9 (1.7)	1 (0.2)	3 (0.6)	0 (0.0)	0 (0.0)	0 (0.0)	15 (2.8)
Soft drinks	672	61 (9.1)	48 (7.1)	1 (0.1)	5 (0.7)	73 (10.9)	0 (0.0)	26 (3.9)	1 (0.1)	6 (0.9)	0 (0.0)	135 (20.1)
Waters	170	0 (0.0)	1 (0.6)	0 (0.0)	0 (0.0)	12 (7.1)	0 (0.0)	7 (4.1)	0 (0.0)	0 (0.0)	0 (0.0)	19 (11.2)
Sauce, dressings, spreads and dips	2347	6 (0.3)	3 (0.1)	0 (0.0)	0 (0.0)	27 (1.2)	0 (0.0)	22 (0.9)	0 (0.0)	0 (0.0)	0 (0.0)	55 (2.3)
Sauces	1767	5 (0.3)	3 (0.2)	0 (0.0)	0 (0.0)	17 (1.0)	0 (0.0)	12 (0.7)	0 (0.0)	0 (0.0)	0 (0.0)	36 (2.0)
Dressings	333	1 (0.3)	0 (0.0)	0 (0.0)	0 (0.0)	10 (3.0)	0 (0.0)	9 (2.7)	0 (0.0)	0 (0.0)	0 (0.0)	18 (5.4)
Spreads and dips	247	0 (0.0)	0 (0.0)	0 (0.0)	0 (0.0)	0 (0.0)	0 (0.0)	1 (0.4)	0 (0.0)	0 (0.0)	0 (0.0)	1 (0.4)

Food category	Total *n* in category	E950	E951	E952	E954	E955	E959	E960	E961	E962	E969	Contains at least one NNS
Snack foods	1002	10 (1.0)	60 (6.0)	2 (0.2)	6 (0.6)	54 (5.4)	0 (0.0)	37 (3.7)	5 (0.5)	1 (0.1)	0 (0.0)	146 (14.6)
Potato crisps	312	2 (0.6)	36 (11.5)	0 (0.0)	0 (0.0)	18 (5.8)	0 (0.0)	14 (4.5)	1 (0.3)	0 (0.0)	0 (0.0)	63 (20.2)
Other crisps or extruded snacks	420	1 (0.2)	20 (4.8)	0 (0.0)	2 (0.5)	28 (6.7)	0 (0.0)	20 (4.8)	3 (0.7)	0 (0.0)	0 (0.0)	57 (13.6)
Popcorn	32	1 (3.1)	1 (3.1)	0 (0.0)	0 (0.0)	0 (0.0)	0 (0.0)	0 (0.0)	0 (0.0)	0 (0.0)	0 (0.0)	2 (6.3)
Other snacks	238	6 (2.5)	3 (1.3)	2 (0.8)	4 (1.7)	8 (3.4)	0 (0.0)	3 (1.3)	1 (0.4)	1 (0.4)	0 (0.0)	24 (10.1)
Sugars, honey and related products	490	2 (0.4)	5 (1.0)	0 (0.0)	1 (0.2)	7 (1.4)	0 (0.0)	10 (2.0)	0 (0.0)	0 (0.0)	0 (0.0)	23 (4.7)
Sweeteners	23	2 (8.7)	5 (21.7)	0 (0.0)	1 (4.3)	6 (26.1)	0 (0.0)	9 (39.1)	0 (0.0)	0 (0.0)	0 (0.0)	21 (91.3)
Sugars or honey or syrups	467	0 (0.0)	0 (0.0)	0 (0.0)	0 (0.0)	1 (0.2)	0 (0.0)	1 (0.2)	0 (0.0)	0 (0.0)	0 (0.0)	2 (0.4)
Total	19915	327 (1.6)	261 (1.3)	32 (0.2)	59 (0.3)	387 (1.9)	1 (0.0)	197 (1.0)	12 (0.1)	10 (0.1)	1 (0.0)	904 (4.5)

E950: acesulfame K; E951: aspartame; E952: cyclamic acid and its sodium or calcium salts; E954: saccharin and its sodium salts; E955: sucralose; E959: neohesperidin DC; E960: stevia; E961: neotame; E962: acesulfame-aspartame salt; E969: advantame.[1]No product in the dataset contained E956 (alitame) or E957 (thaumatin)

Supplementary Table 2. – Prevalence [*n* (%)] of use of different low-calorie sweeteners (LCSs) by food category.

**Food category**	**Total n in category**	**E953**	**E965**	**E966**	**E967**	**E968**	**E420**	**E421**	**Luo han guo**	**Contains at least one LCS**
Bread and bakery products	1774	9 (0.5)	22 (1.2)	0 (0.0)	0 (0.0)	1 (0.1)	99 (5.6)	3 (0.2)	1 (0.1)	125 (7.0)
Biscuits	1085	4 (0.4)	15 (1.4)	0 (0.0)	0 (0.0)	1 (0.1)	25 (2.3)	0 (0.0)	0 (0.0)	40 (3.7)
Breads	181	0 (0.0)	0 (0.0)	0 (0.0)	0 (0.0)	0 (0.0)	1 (0.6)	0 (0.0)	0 (0.0)	1 (0.6)
Cakes and other bakery items	508	5 (1.0)	7 (1.4)	0 (0.0)	0 (0.0)	0 (0.0)	73 (14.4)	3 (0.6)	1 (0.2)	84 (16.5)
Cereal and grain products	2826	5 (0.2)	9 (0.3)	0 (0.0)	0 (0.0)	0 (0.0)	79 (2.8)	2 (0.1)	2 (0.1)	94 (3.3)
Breakfast cereals	466	0 (0.0)	3 (0.6)	0 (0.0)	0 (0.0)	0 (0.0)	0 (0.0)	0 (0.0)	2 (0.4)	5 (1.1)
Cereals and nut-based bars	118	5 (4.2)	5 (4.2)	0 (0.0)	0 (0.0)	0 (0.0)	6 (5.1)	0 (0.0)	0 (0.0)	13 (11.0)
Noodles	1127	0 (0.0)	1 (0.1)	0 (0.0)	0 (0.0)	0 (0.0)	72 (6.4)	2 (0.2)	0 (0.0)	75 (6.7)
Pasta, rice or cous cous	655	0 (0.0)	0 (0.0)	0 (0.0)	0 (0.0)	0 (0.0)	1 (0.2)	0 (0.0)	0 (0.0)	1 (0.2)
Other cereal products	460	0 (0.0)	0 (0.0)	0 (0.0)	0 (0.0)	0 (0.0)	0 (0.0)	0 (0.0)	0 (0.0)	0 (0.0)
Confectionery	1710	36 (2.1)	70 (4.1)	7 (0.4)	25 (1.5)	11 (0.6)	225 (13.2)	54 (3.2)	1 (0.1)	304 (17.8)
Chocolates	735	0 (0.0)	11 (1.5)	6 (0.7)	0 (0.0)	0 (0.0)	39 (5.3)	0 (0.0)	0 (0.0)	56 (7.6)
Sweets	658	20 (3.0)	5 (0.8)	1 (0.2)	0 (0.0)	4 (0.6)	119 (18.1)	14 (2.1)	0 (0.0)	151 (22.9)
Chewing gums	57	6 (10.5)	42 (73.7)	0 (0.0)	24 (42.1)	2 (3.5)	46 (80.7)	39 (68.4)	1 (1.8)	51 (89.5)
Cough lollies	61	7 (11.5)	8 (13.1)	0 (0.0)	1 (1.6)	1 (1.6)	8 (13.1)	1 (1.6)	0 (0.0)	25 (41.0)
Jellies	199	3 (1.5)	4 (2.0)	0 (0.0)	0 (0.0)	4 (2.0)	13 (6.5)	0 (0.0)	0 (0.0)	21 (10.6)
Convenience foods	1171	0 (0.0)	3 (0.3)	0 (0.0)	0 (0.0)	0 (0.0)	36 (3.1)	0 (0.0)	0 (0.0)	38 (3.2)
Dairy	1611	0 (0.0)	5 (0.3)	0 (0.0)	0 (0.0)	1 (0.1)	9 (0.6)	0 (0.0)	0 (0.0)	15 (0.9)
Edible oils and oil emulsions	459	0 (0.0)	0 (0.0)	0 (0.0)	0 (0.0)	0 (0.0)	1 (0.2)	0 (0.0)	0 (0.0)	1 (0.2)
Eggs	74	0 (0.0)	0 (0.0)	0 (0.0)	0 (0.0)	0 (0.0)	1 (1.4)	0 (0.0)	0 (0.0)	1 (1.4)
Seafood and seafood products	740	0 (0.0)	0 (0.0)	0 (0.0)	1 (0.1)	0 (0.0)	27 (3.6)	0 (0.0)	0 (0.0)	27 (3.6)
Fruit and vegetables	2525	0 (0.0)	1 (0.0)	0 (0.0)	0 (0.0)	0 (0.0)	27 (1.1)	1 (0.0)	0 (0.0)	28 (1.1)
Fruits	460	0 (0.0)	0 (0.0)	0 (0.0)	0 (0.0)	0 (0.0)	0 (0.0)	0 (0.0)	0 (0.0)	0 (0.0)
Herbs and spices	586	0 (0.0)	0 (0.0)	0 (0.0)	0 (0.0)	0 (0.0)	5 (0.9)	1 (0.2)	0 (0.0)	6 (1.0)
Jam and marmalade	222	0 (0.0)	0 (0.0)	0 (0.0)	0 (0.0)	0 (0.0)	6 (2.7)	0 (0.0)	0 (0.0)	6 (2.7)
Nuts and seeds	431	0 (0.0)	0 (0.0)	0 (0.0)	0 (0.0)	0 (0.0)	1 (0.2)	0 (0.0)	0 (0.0)	1 (0.2)
Vegetables	826	0 (0.0)	1 (0.1)	0 (0.0)	0 (0.0)	0 (0.0)	15 (1.8)	0 (0.0)	0 (0.0)	15 (1.8)
Meat and meat alternatives	763	0 (0.0)	0 (0.0)	0 (0.0)	0 (0.0)	0 (0.0)	9 (1.2)	1 (0.1)	0 (0.0)	10 (1.3)
Non-alcoholic beverages	2423	1 (0.0)	0 (0.0)	0 (0.0)	3 (0.1)	14 (0.6)	3 (0.1)	0 (0.0)	3 (0.1)	23 (0.9)
Coffee, tea and hot chocolates	915	0 (0.0)	0 (0.0)	0 (0.0)	3 (0.3)	0 (0.0)	0 (0.0)	0 (0.0)	0 (0.0)	3 (0.3)
Cordials	94	0 (0.0)	0 (0.0)	0 (0.0)	0 (0.0)	0 (0.0)	0 (0.0)	0 (0.0)	1 (1.1)	1 (1.1)
Electrolyte and energy drinks	45	0 (0.0)	0 (0.0)	0 (0.0)	0 (0.0)	2 (4.4)	0 (0.0)	0 (0.0)	1 (2.2)	2 (4.4)
Fruit and vegetable juices	527	0 (0.0)	0 (0.0)	0 (0.0)	0 (0.0)	0 (0.0)	0 (0.0)	0 (0.0)	0 (0.0)	0 (0.0)
Soft drinks	672	1 (0.1)	0 (0.0)	0 (0.0)	0 (0.0)	9 (1.3)	3 (0.4)	0 (0.0)	1 (0.1)	14 (2.1)
Waters	170	0 (0.0)	0 (0.0)	0 (0.0)	0 (0.0)	3 (1.8)	0 (0.0)	0 (0.0)	0 (0.0)	3 (1.8)
Sauce, dressings, spreads and dips	2347	0 (0.0)	1 (0.0)	0 (0.0)	0 (0.0)	1 (0.0)	22 (0.9)	0 (0.0)	0 (0.0)	23 (1.0)
Sauces	1767	0 (0.0)	0 (0.0)	0 (0.0)	0 (0.0)	0 (0.0)	20 (1.1)	0 (0.0)	0 (0.0)	20 (1.1)
Dressings	333	0 (0.0)	0 (0.0)	0 (0.0)	0 (0.0)	0 (0.0)	1 (0.3)	0 (0.0)	0 (0.0)	1 (0.3)
Spreads and dips	247	0 (0.0)	1 (0.4)	0 (0.0)	0 (0.0)	1 (0.4)	1 (0.4)	0 (0.0)	0 (0.0)	2 (0.8)
Snack foods	1002	1 (0.1)	0 (0.0)	0 (0.0)	0 (0.0)	0 (0.0)	30 (3.0)	0 (0.0)	0 (0.0)	31 (3.1)
Potato crisps	312	0 (0.0)	0 (0.0)	0 (0.0)	0 (0.0)	0 (0.0)	0 (0.0)	0 (0.0)	0 (0.0)	0 (0.0)
Other crisps or extruded snacks	420	0 (0.0)	0 (0.0)	0 (0.0)	0 (0.0)	0 (0.0)	10 (2.4)	0 (0.0)	0 (0.0)	10 (2.4)

Food category	Total n in category	E953	E965	E966	E967	E968	E420	E421	Luo han guo	Contains at least one LCS
Popcorn	32	1 (3.1)	0 (0.0)	0 (0.0)	0 (0.0)	0 (0.0)	1 (3.1)	0 (0.0)	0 (0.0)	2 (6.3)
Other snacks	238	0 (0.0)	0 (0.0)	0 (0.0)	0 (0.0)	0 (0.0)	19 (8.0)	0 (0.0)	0 (0.0)	19 (8.0)
Sugars, honey and related products	490	0 (0.0)	0 (0.0)	0 (0.0)	0 (0.0)	10 (2.0)	0 (0.0)	0 (0.0)	2 (0.4)	10 (2.0)
Sweeteners	23	0 (0.0)	0 (0.0)	0 (0.0)	0 (0.0)	10 (43.5)	0 (0.0)	0 (0.0)	2 (8.7)	10 (43.5)
Sugars or honey or syrups	467	0 (0.0)	0 (0.0)	0 (0.0)	0 (0.0)	0 (0.0)	0 (0.0)	0 (0.0)	0 (0.0)	0 (0.0)
Total	19915	52 (0.3)	111 (0.6)	7 (0.0)	29 (0.1)	38 (0.2)	568 (2.9)	61 (0.3)	9 (0.0)	730 (3.7)

E953: isomalt; E965: maltitol; E966: lactitol; E967: xylitol; E968: erythritol; E420: sorbitol; E421: mannitol.
